# Low-power transcutaneous current stimulator for wearable applications

**DOI:** 10.1186/s12938-017-0409-9

**Published:** 2017-10-03

**Authors:** David Karpul, Gregory K. Cohen, Gaetano D. Gargiulo, André van Schaik, Sarah McIntyre, Paul P. Breen

**Affiliations:** 1 The MARCS Institute for Brain, Behaviour and Development, Western Sydney University, Bullecourt Avenue, Milperra, Sydney, Australia; 2 Division of Neurology, Department of Medicine, University of Cape Town, Main Road, Rondebosch, Cape Town, South Africa; 3 Neuroscience Research Australia, Barker St, Randwick, Sydney, Australia

**Keywords:** Wearable, Low-power, Low-cost, Transcutaneous electrical nerve stimulation, Stochastic resonance, Peripheral sensory neuropathy

## Abstract

**Background:**

Peripheral neuropathic desensitization associated with aging, diabetes, alcoholism and HIV/AIDS, affects tens of millions of people worldwide, and there is little or no treatment available to improve sensory function. Recent studies that apply imperceptible continuous vibration or electrical stimulation have shown promise in improving sensitivity in both diseased and healthy participants. This class of interventions only has an effect during application, necessitating the design of a wearable device for everyday use. We present a circuit that allows for a low-power, low-cost and small form factor implementation of a current stimulator for the continuous application of subthreshold currents.

**Results:**

This circuit acts as a voltage-to-current converter and has been tested to drive + 1 to − 1 mA into a 60 k$\Omega$ load from DC to 1 kHz. Driving a 60 k$\Omega$ load with a 2 mA peak-to-peak 1 kHz sinusoid, the circuit draws less than 21 mA from a 9 V source. The minimum operating current of the circuit is less than 12 mA. Voltage compliance is ± 60 V with just 1.02 mA drawn by the high voltage current drive circuitry. The circuit was implemented as a compact 46 mm × 21 mm two-layer PCB highlighting its potential for use in a body-worn device.

**Conclusions:**

No design to the best of our knowledge presents comparably low quiescent power with such high voltage compliance. This makes the design uniquely appropriate for low-power transcutaneous current stimulation in wearable applications. Further development of driving and instrumentation circuitry is recommended.

## Background

Peripheral neuropathic desensitization is a common problem that can be caused by diabetes, stroke, alcoholism, HIV, aging and many other conditions. It is estimated that 20–30 million people worldwide suffer symptomatic diabetic neuropathy [1]. Reduced peripheral sensation is seen as a normal part of the ageing process [2]. In South Africa, as many as 1.8 million people suffer HIV-related peripheral neuropathy [3].

Length-dependent poly-neuropathy, the most common form of peripheral neuropathy, causes reduced tactile sensation primarily in the extremities, which dramatically impacts quality of life through reduced sensory feedback and motor control. Currently there is little to no treatment that improves peripheral sensitivity in these populations [4].

A class of potential interventions applying imperceptible vibration or electrical stimulation has shown promise in improving peripheral sensitivity in both people with peripheral neuropathy and healthy participants. The interventions apply a signal, usually vibration (e.g. [5]) or electrical current (e.g. [6]), at either the target site (e.g. [7]), or proximal to the target site (e.g. [8]). Various performance parameters have been shown to improve, such as tactile sensitivity, balance, gait, and performance in dexterous tasks [9–11]. Subthreshold electrical stimulation interventions have also been implemented to improve balance through vestibular stimulation (e.g. [12–15]).

The intervention usually takes the form of a continuous signal, typically band-limited white noise, which is applied at amplitudes between 60 and 90% of perception threshold. The interventions have shown no ability to have lasting effects once removed, thus necessitating a wearable version for continuous use. This methodology is contrary to previous interventions that applied suprathreshold signals in an attempt to create lasting effects, such as TENS [16].

Theoretically this class of interventions work through the mechanism of stochastic facilitation, whereby the resting potentials of underlying tactile nerves are altered by the intervention signal and thus become more likely to fire under near threshold conditions [17]. A second possible mechanism may be an increase in inter-spike synchronization allowing for easier detection of a signal, either at the dorsal root junction or more centrally [18].

Studies have not yet progressed to experiments outside of laboratory conditions but there is scope to start investigating more long-term application and to adapt the interventions for the practical considerations of everyday use. While the majority of previous experiments investigated the application of a vibratory intervention, the electrical stimulation variant would in theory allow a smaller, cheaper, and lower power solution.

Two factors cause the design of a low-power, continuous, current stimulator for human applications to be challenging. First, driving small currents into large loads requires a very high output impedance current drive. This can be solved using an improved Howland current generator [19]. Secondly, the load itself, two conductive electrodes attached across a limb, has a very large series resistive component at low frequencies, necessitating substantial voltage compliance to drive current into the limb if an arbitrary signal is required.

**Fig. 1:**
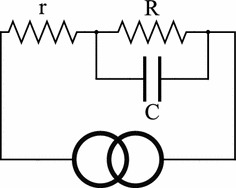
Effective network impedance of two electrodes connected to a human appendage driven by a current source. r is typically in the order of 2 k$\Omega$, and can be thought to roughly represent the resistance of the limb itself. R and C are usually in the order of 20–60 k$\Omega$ and 30–600 nF and represent the resistance and capacitance of the electrode connection to the skin

The possible magnitudes of the impedance connected to a current stimulator have a dramatic influence on the design specifications of the device. Bîrlea et al. performed a study that investigated participants who wore electrodes for seven days without removal and monitored the changes in impedance over time [20]. The impedance formed between the stimulation electrodes was modelled as a network of a single small resistor (r) in series with the parallel combination of a large resistor (R) and capacitor (C) (Fig 1). r is typically in the order of 2 k$\Omega$, and can be thought to represent the resistance of the limb itself. R and C are usually in the order of 20–60 k$\Omega$ and 30–600 nF and represent the resistance and capacitance of the electrode connection to the skin respectively. This model of R, r and C accurately fits experimental impedance measures of different electrode types [21]. At high frequencies and pulsatile applications, C effectively shorts out R and thus r dominates the impedance of the network. However, close to DC conditions, C is open circuit and R dominates, resulting in a high-impedance that requires large voltages to achieve the desired currents. An arbitrary signal current pump would need to be able to drive a worst-case load of 60 k$\Omega$. To drive a 60 k$\Omega$ load, 60 V is needed for every mA of current, thus requiring 120 V in total to facilitate + 1 to − 1 mA range.

This high voltage requirement can be solved by using a switching boost converter to generate a high voltage power supply from a battery, and by using high voltage op-amps in the design of the Howland current pump. However, switching converters, which often use inductors, are noisy, often draw excessive quiescent current, and are difficult to implement, often not producing the expected output. High voltage op-amps are expensive and draw larger quiescent currents than their low voltage counterparts.

Here we present a solution to these problems, specifically tailored for continuous subthreshold transcutaneous neural stimulation.

## Design of circuitry for a wearable current stimulator

### Design specifications

The device needs to be sufficiently compact and lightweight so that it can be worn in every day circumstances. It should be able operate continuously for at least 10 h without the need for recharging or replacing batteries, and it should be capable of applying electrical stimulation consistent with that used in previous studies (e.g. [22]). 10 h was selected as this is the upper limit of the average workday, and would allow interventions to be investigated for continuous effect over the periods where improved sensation would have the most impact on function. Consequently, the proposed circuit needs the following attributes:

- Capable of driving a continuous current of + 1 to − 1 mA under worst-case load conditions.
- Have a frequency range of at least 0–1 kHz.
- Draw sufficiently low power so that 10 h of operation can be achieved on a single battery charge, without the need for large cumbersome batteries.
- Consist of parts with sufficiently small form factors such that the overall device is compact and practical.
- Have a low manufacturing cost and be easy to implement.

### Design of the high voltage power supply unit (HVPSU)

The worst-case load impedance can be estimated as 60 k$\Omega$ when driving DC currents. This necessitates a HVPSU voltage of at least − 60 to + 60 V, given the minimum output current requirements of + 1 to − 1 mA. The “inverted-reference” design of the current pump presented below allows for half this voltage to be used to achieve the same output current, necessitating a HVPSU capable of producing 60 V when under load.

In theory, any boost converter with a sufficiently low quiescent current, capable of delivering more than 1 mA at 60 V from battery packs, would be appropriate. Of course, the HVPSU needs to supply additional current to power the subsequent circuitry.

**Fig. 2:**
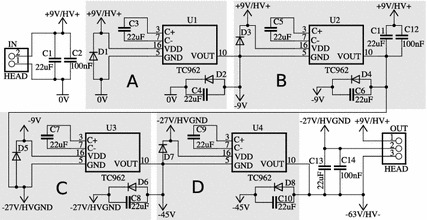
Design of a 9–72 V converter using cascaded voltage inverters. The positive terminal of the input supply becomes the high voltage output, and the most negative output of the inverters, − 63 V, is 72 V below the positive terminal and forms the negative output of the high voltage supply. The blocks A, B, C and D are each independent voltage inverters capable of inverting a maximum of 18 V

**Fig. 3:**
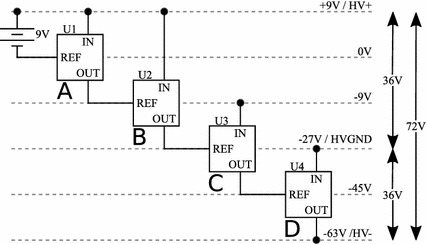
Explanatory diagram of the HVPSU. Four inverters, U1–U4, convert 9 V from the battery to a 72 V power supply with a midpoint tap at 36 V. Each inverter takes the difference between REF and IN as an input and inverts it below the REF input. The inverters can accept a maximum of 18 V as an input. The labels A, B, C and D correspond to the circuitry blocks with the same labels in Fig. 2

Our design uses a cascaded series of TC962 voltage inverters to construct the desired HVPSU (Figs. 2, 3). These inverters offer low quiescent current, are stable and efficient. The TC962 is a pin-for-pin replacement for the industry standard voltage inverter: the ICL7662. While the two chips are similar in most respects, the TC962 has a lower output impedance, which improves the performance of the circuit. In theory one could replace the TC962 with ICL7662 if low output impedance was not desired.

In this application, we used a 9 V battery and four inverters to achieve an HVPSU voltage of 72 V. The 9 V battery is first inverted to create − 9 V using a TC962 in its standard configuration (shown in block A of Figs. 2, 3). The new total available voltage of 18 V above the − 9 V rail is then inverted around the − 9 V rail to create − 27 V (shown in block B of Figs. 2, 3). The total 36 V available is now too large to apply to a further TC962, which only allows an input voltage of 18 V. The next stage inverts the − 9 V rail around the lowest available rail of − 27 V to create − 45 V (shown in block C of Figs. 2, 3). Finally the − 27 V rail is inverted around the − 45 V rail to create − 63 V (shown in block D of Figs. 2, 3). Treating the positive terminal of the battery as V+ and the most negative voltage available as V−, a total of 72 V is now available (9 V − (− 63 V) = 72 V). The − 27 V rail is midway between V+ and V− and can act as a pseudo split-rail 0 V for subsequent circuitry (9 V − (− 27 V) = 36 V).

The actual voltage achieved will depend on the current drawn by the subsequent current pump due to the output impedance of the HVPSU. 22 μF capacitors, as opposed to the standard design using 10 μF capacitors, were used throughout the design to reduce the final output impedance. Protection diodes were also added to each stage to prevent over-voltage inputs.

As the output voltage is now eight times the input, and power is conserved throughout, the current drawn from the output of the HVPSU will be scaled up when traced back to the battery. If 1 mA is drawn from the HVPSU, then 8 mA will be drawn from the battery. This emphasizes the importance of the low quiescent current in the current drive circuitry. This will hold true for any boost HVPSU.

### Current source design

**Fig. 4:**
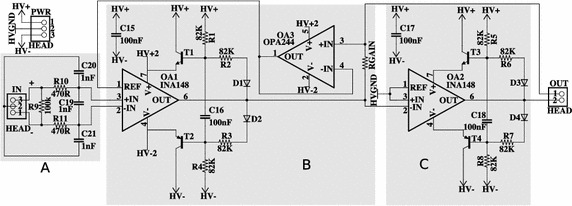
Schematic for a high-voltage, low-power transcutaneous current stimulator for wearable applications. Block A is a differential low-pass filter. Block B is a modified Howland current pump. The circuit takes advantage of bootstrapping transistors to enable low voltage differential amplifiers to operate at high voltage. Furthermore, the addition of an inverting amplifier (Block C) driving the reference electrode, allows the full supply voltage to be applied over the load in both directions, halving the requirement for the supply voltage

Figure 4 shows the design of the high voltage current pump (HVCP). A differential input voltage applied to the positive and negative inputs of OA1 (via a differential low-pass filter, block A in Fig. 4), at the “IN” header, and is converted to a proportional current via the gain control resistor RGain:

1 $$\begin{aligned} Iload = (Vin_+ - Vin_-) /RGain. \end{aligned}$$

This current is output via one electrode connection at pin 1 of the “OUT” header, and returns at electrode connection pin 2 of the “OUT” header. OA1 is a difference amplifier with internal laser-trimmed resistors such that OA1 and OA3 form the modified Howland current pump covered in detail in [19] (block B in Fig. 4). The differential low-pass filter is added to reduce high frequency steps created by digital controllers potentially used to drive the HVCP.

The electrode connection at pin 2 of the “OUT” header would typically just be kept at 0 V, or in this case HVGND. Since the current pump does not require feedback from this reference electrode, we are free to manipulate its voltage to improve compliance. Here we have inverted the positive drive signal via OA2 and applied it to the reference electrode (block C in Fig. 4). This allows the full voltage of the power supply to be applied positively and negatively over the load in a similar fashion to an H-bridge motor driver. This halves the maximum voltage required from the HVPSU for the circuit to achieve a desired alternating current through a specific load. OA2 is a unity gain inverter.

T1 to T4 bootstrap the op-amps’ power supplies as described in [23] and [24]. The op-amps’ power rails are adjusted as needed by the circuit and only ever see the portion of the supply voltage they require at that instant, linking their output voltage to the supply voltage. This allows the use of low voltage op-amps for high voltage applications simply by adding low-cost, high-voltage transistors (in this case BC546 and BC556 transistors). Any transistors with sufficient frequency, current gain, and voltage tolerances will suffice.

The bootstrapping solution creates a new problem in that the inputs of the op-amp can now fall well outside the power supply at any one time, even though the differential input voltage may be small.

Consequently, both OA1 and OA2 need to be specialized differential amplifiers capable of handling common mode inputs beyond their supply rails. Various commercially available amplifiers exist with this feature. Here we use a Texas Instruments INA148 which can handle ± 200 V common-mode difference and draws a quiescent current of only 260 μA, making it ideal for this application. In contrast, a high-voltage op-amp such as the OPA454, which operates to 100 V, draws 3–4 mA quiescent current.

OA3 provides the required feedback voltage for the HVCP without drawing current from the load. It is vital that this op-amp has a high input impedance and it is preferable that the op-amp draws low quiescent current and has similar supply rail limitations to OA1 (in this case an OPA244). OA3 does not need independent bootstrapping, nor does it need to handle common-mode signals beyond its rails, as its input is only slightly different to OA1’s output, so OA3 can share OA1’s floating supply.

## Results

The HVPSU and HVCP test circuits were designed as two separate printed circuit boards (PCB’s), each with additional voltage test points and ammeter insertion points included in the design. No attempt was made to minimize the size of these circuits in this initial test stage, as ease of access to signals was required for characterization.

### HVPSU results

**Fig. 5:**
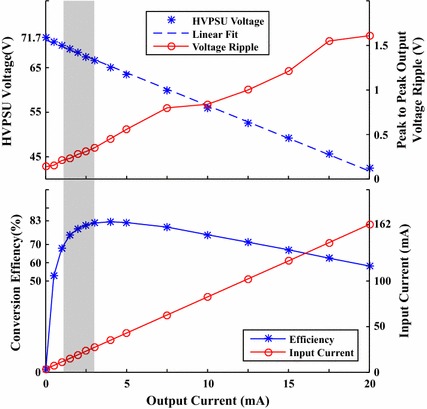
Various HVPSU parameters plotted against output current. Top: shows how the output voltage drops linearly with current draw, consistent with an output impedance of 1.47 k$\Omega$. The linear fit has an $R^2 > 0.998$. The switching converters produce voltage ripple on the output that increases with current drawn. Bottom: shows the efficacy of the circuit with respect to output current. For low currents the quiescent current of the circuit dominates the output power. At higher currents the loss over the effective output impedance dominates. Current drawn from the battery is also shown to rise at approximately eight times the high voltage output current in accordance with theory. The typical operating current range of the subsequent HVCP is shown as the shaded region

Figure 5 shows the output voltage of the HVPSU, when supplied with 9 V, at various current draws. Current draw and efficiency is also plotted.

When drawing 20 mA from the HVPSU, the current output of the first voltage inverter is 80 mA, the maximum rated current for a TC962. The circuit was not tested beyond this limiting point.

The HVPSU produced 71.7 V with no load. Progressively increasing the current load on the HVPSU up to 20 mA showed a near linear reduction in voltage consistent with a constant output impedance of 1.470 k$\Omega$.

### HVCP results

**Fig. 6:**
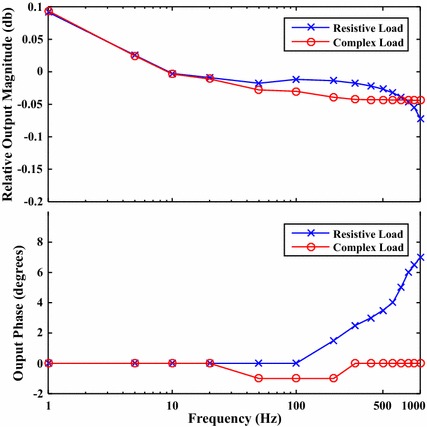
Graphs of relative output magnitude and output phase shift at various frequencies. The resistive load was 60 k$\Omega$. The complex load was a 58 k$\Omega$ resistor with a 30 nF capacitor in parallel, both in series with a 2 k$\Omega$ resistor in the configuration of Fig. 1. Both loads were tested for a constant drive amplitude of 2 mA peak to peak. $relative\_output = 10*\log _{10}(Amplitude/Amplitude\_at\_10\_Hz)$. The output had minimal attenuation and phase response, especially at lower frequencies

The HVCP, supplied by the HVPSU, was evaluated using both a 60 k$\Omega$ resistive load as a worst-case impedance test, and a complex load in the same form as Fig. 1, with R = 58 k$\Omega$, r = 2 k$\Omega$, and C = 30 nF. Figure 6 shows the output gain amplitude and phase offset at various frequencies when driving a maximum of + 1 to − 1 mA sinusoid. The circuit was also tested using various simple resistive loads down to short circuit conditions.

The circuit was able to drive the required current over the entire frequency range with negligible phase offset and no clipping, in accordance with Eq. 1 under all load conditions.

The current consumption of the circuit was measured under various conditions and is shown in Table 1.

**Table 1:** HVPSU and HVCP current draw under various signal drive conditions for a load of 60 k$\Omega$

Load	0 mA	1 mA	− 1 mA	2 mA^a^	2 mA^a^
Current	DC	DC	DC	100 Hz	1 kHz
HVCP only (mA)	1.02	2.53	3.07	2.15	2.14
9 V supply current (mA)	11.69	25.6	25.9	20.6	20.5

^a^Peak to peak

**Fig. 7:**
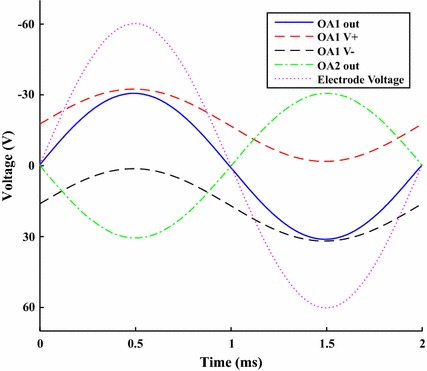
Test voltages of HVCP. The traces demonstrate: (1) how the bootstrapping of OA1’s supply lines allow it to output a range beyond its usual limits, and (2) how inverting the current pump output onto the second electrode allows for the full 60 V to be applied bidirectionally over the electrodes. Test conditions were driving a 2 mA peak to peak sinusoidal current into a 60 k$\Omega$ load at 500 Hz

Figure 7 shows the output of OA1 driving a sinusoidal signal of 60 V peak to peak, without its supply rails exceeding the maximum rating for that chip of 36 V difference. It further shows the full + 60 and − 60 V being applied across the load terminals.

**Fig. 8:**
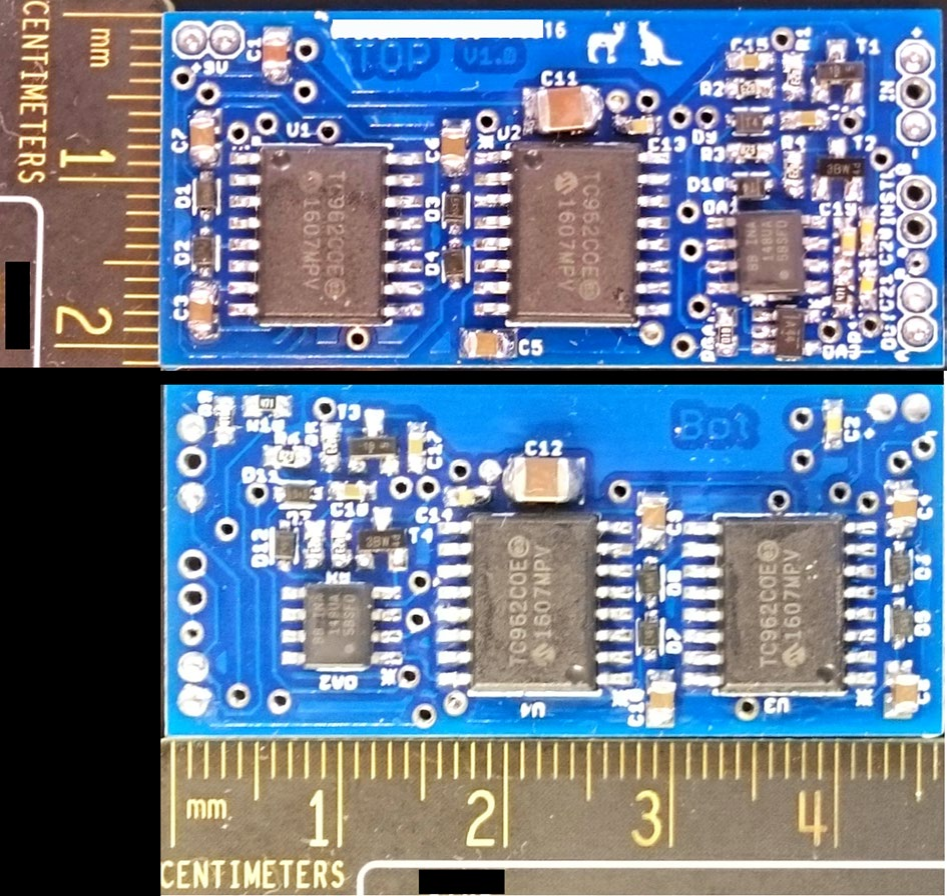
Top and bottom views of the compact version of low-power transcutaneous current stimulator for wearable applications

The two circuits were redesigned as a single small form factor, two-layer PCB (Fig. 8). In this design the smallest package component versions available were used, and all test points were removed. The final PCB design measured 46 mm × 21 mm. This circuit performed as expected, with the change in form factor having no impact on performance.

The total cost of the parts for this small version from online vendors is less than 35 USD when purchased in low quantities.

## Discussion

The HVPSU had poor efficiency for currents below 0.5 mA, but had efficiencies above 75% for currents between 1.5 and 10 mA. It is important to consider quiescent current when looking at the efficiency outcomes. The circuit only draws a quiescent current of  3.6 mA when under no load. Consequently, when supplying power in the same range of the quiescent power, the subsequent efficiency calculation will be very poor, around 50%. This improves as more power is drawn, but will peak when the load reaches the internal impedance of the HVPSU.

One can subtract the quiescent power before calculating efficiency to get a metric of conversion efficacy alone. Doing this, the efficiency is then 100% at no load and steadily decreases to 91.7% at 4 mA and 78% at 10 mA and then follows the original efficiency curve.

The HVPSU showed an increase in voltage ripple with current draw. As more current is drawn this voltage ripple would act to reduce minimum guaranteed output voltage of the HVPSU, and thus compliance of the HVCP. The operating range of the subsequent HVCP however keeps the HVPSU voltage ripple under 0.5 V, which allows the HVCP to operate unaffected.

The HVCP only draws 1.02 mA when under no load, far superior when compared to the current draw of just one high voltage op-amp. The worst 9 V (battery) current consumption of 25.9 mA occurred when driving a DC current of − 1 mA into the load. The full circuit would therefore require a battery of at least 260 mAh at 9 V in order to operate for 10 h without recharging or replacement under worst-case conditions. A standard 9V battery has a capacity of between 300 and 500 mAh. Dividing this by the worst-case current consumption of 25.9 mA yields a charge life of 11.6–19.3 h.

The deviation in output magnitude and phase at frequencies above 500 Hz for the resistive load, did not occur when testing with small loads, or the complex load, requiring smaller voltages to drive the required currents. Changing the values of the stabilizing capacitors C16 and C18 or the values of the transistor biasing resistors R1 to R8 had no effect on this phenomenon. The effect is likely caused by the op-amp supply bootstrapping. This creates very large common mode swings for both the supply voltage, and the relative input voltages to OA1 and OA2 under these conditions. The data-sheet for the INA148 indicates that both the common mode rejection ratio and the power supply rejection ratio start to fall as these signals approach 1 kHz. This problem is unlikely to affect actual stimulation applications, as the impedance of a typical skin electrode pair drops quickly with frequency. This means that the high frequency components will not induce these large common mode swings.

The “inverted reference” design was shown to be stable and did not impede the performance of the Howland current pump. However, for small loads below 2 k$\Omega$ high frequency oscillations may occur. While this is unlikely to occur in practice, the problem can be solved by inserting low pass filter inline with the input of OA2. This inverted reference configuration also improves the safety of the circuit, as the largest voltage in the device is now 72 V as opposed to 144 V if electrode B were held at HVGND.

To our knowledge, the only example of a stimulator designed with similar application in mind was created by Yamamoto et al. [12, 14, 15]. We also compare three wireless stimulators which have similar design constraints to those used here [25–27]. While there are many differences created through differing needs of the end application, it is vital to note that these wireless stimulators are designed for pulsatile applications. These are much more common, but cannot be used for continuous signal stimulation needed for subthreshold interventions.

It is important to consider the power consumption of control circuitry not included in our design. Many appropriate microcontrollers are available that do not consume significant power, and have a small form factor. For example, the PIC24FJ128GC006, which has built in DAC’s, ADC’s and analog circuitry. This chip consumes less than 13 mW at 8 MHz. The results of the comparison are contained in Table 2. We have added the weight and dimensions of a standard 9 V battery (46 g and 48.8 mm × 26 mm × 16.9 mm) to our design in the figures of Table 2.

**Table 2:** Comparison to designs with similar constraints in the literature

	Karpul et al. [21]	Yamamoto et al. [12, 14, 15]	Wang et al. [27]	Farahmand et al. [25]	Jovičić et al. [26]
Voltage compliance (V)	± 72	± 10^a^	± 60	23	85
Power consumption (mW)	233	312^a^	720	51.2	> 700
Use duration (h)	> 10	> 24^a^	8	Unknown	Unknown
Current output (mA)	± 1	± 1	60	0.4	70
Volume (cm^3^)	28.2	210.1	127.5	90	52.5
Weight (g)	52	200	85	60	45
Signal type	Continuous	Continuous	Pulsatile	Pulsatile	Pulsatile

^a^Approximate values based on information directly from the author

The table indicates that our design has a smaller form factor and higher compliance than those with which it is compared. The power consumption of the circuit is also superior to those designs with higher compliance (greater than 23 V).

## Conclusions

Here we presented a current stimulator designed to overcome the challenges associated with continuous, low-power transcutaneous current stimulation for the improvement of peripheral sensitivity. We have shown the circuit to perform within specifications under worst-case load conditions. What makes the design most unique is its low power consumption, high voltage compliance, and small form factor making it specifically appropriate for wearable applications.

To the best of our knowledge, this is the first paper to demonstrate a full design specifically targeting subthreshold stochastic stimulation in wearable applications, with high voltage compliance, continuous-signal output, and sufficiently low power operation to be used in wearable applications. A list of specifications to be met in this application is proposed. The design adds to previous work by including an inverting reference to double the voltage compliance, a differential input filter to reduce noise from DAC’s, a change of various components to reduce current consumption and ensure the circuit is appropriate for the application, and the inclusion of a low quiescent current HVPSU that is compact and simple to construct. Finally, a characterization specifically focusing on aspects that apply to the intended application is presented.

The next step is to allow for the driving and instrumentation of the circuit using additional low power analog and digital circuitry. Finally the circuit must be tested on a human limb analog and eventually on human participants.

## Abbreviations

HVPSU: high voltage power supply
HVCP: high voltage current pump
PCB: printed circuit board
DAC: digital to analog converter
ADC: analog to digital converter
